# Molecular Profiles of Amyloid-β Proteoforms in Typical and Rapidly Progressive Alzheimer’s Disease

**DOI:** 10.1007/s12035-021-02566-9

**Published:** 2021-10-07

**Authors:** Aneeqa Noor, Saima Zafar, Mohsin Shafiq, Neelam Younas, Anna Siegert, Florian A. Mann, Sebastian Kruss, Matthias Schmitz, Hassan Dihazi, Isidre Ferrer, Inga Zerr

**Affiliations:** 1 Clinical Department of Neurology, University Medical Center Göttingen and the German Center for Neurodegenerative Diseases (DZNE), Robert-Koch-Straße 40, 37075 Göttingen, Germany; 2 Biomedical Engineering and Sciences Department, School of Mechanical and Manufacturing Engineering (SMME), National University of Sciences and Technology (NUST), Bolan Road, Islamabad, H-12, 44000 Pakistan; 3 Institute of Neuropathology, University Medical Center Hamburg-Eppendorf, Martinistraße 52, 20246 Hamburg, Germany; 4 Institute of Physical Chemistry, Georg-August University, Tammannstraße 6, 37077 Göttingen, Germany; 5 Department of Nephrology and Rheumatology, Georg-August University, University Medical Center Göttingen, Robert-Koch-Straße 40, 37075 Göttingen, Germany; 6 Department of Pathology and Experimental Therapeutics, University of BarcelonaCIBERNEDBellvitge University Hospital (IDIBELL), Carrer de la Feixa Llarga, 08907 Hospitalet de Llobregat, Spain

**Keywords:** Proteoforms, Aβ, Rapidly progressive, Alzheimer’s disease, Top-down proteomics

## Abstract

The molecular determinants of atypical clinical variants of Alzheimer’s disease, including the recently discovered rapidly progressive Alzheimer’s disease (rpAD), are unknown to date. Fibrilization of the amyloid-β (Aβ) peptide is the most frequently studied candidate in this context. The Aβ peptide can exist as multiple proteoforms that vary in their post-translational processing, amyloidogenesis, and toxicity. The current study was designed to identify these variations in Alzheimer’s disease patients exhibiting classical (sAD) and rapid progression, with the primary aim of establishing if these variants may constitute strains that underlie the phenotypic variability of Alzheimer’s disease. We employed two-dimensional polyacrylamide gel electrophoresis and MALDI-ToF mass spectrometry to validate and identify the Aβ proteoforms extracted from targeted brain tissues. The biophysical analysis was conducted using RT-QuIC assay, confocal microscopy, and atomic force microscopy. Interactome analysis was performed by co-immunoprecipitation. We present a signature of 33 distinct pathophysiological proteoforms, including the commonly targeted Aβ_40_, Aβ_42_, Aβ_4-42_, Aβ_11-42_, and provide insight into their synthesis and quantities. Furthermore, we have validated the presence of highly hydrophobic Aβ seeds in rpAD brains that seeded reactions at a slower pace in comparison to typical Alzheimer’s disease. In vitro and in vivo analyses also verified variations in the molecular pathways modulated by brain-derived Aβ. These variations in the presence, synthesis, folding, and interactions of Aβ among sAD and rpAD brains constitute important points of intervention. Further validation of reported targets and mechanisms will aid in the diagnosis of and therapy for Alzheimer’s disease.

**Supplementary Information:**

The online version contains supplementary material available at 10.1007/s12035-021-02566-9.

## Background

The Aβ peptide is one of the thirty amyloidogenic proteins known to cause diseases in humans [1]. It has been conventionally defined as a 42-residue peptide produced through the sequential cleavage of amyloid precursor protein (APP). Aβ peptide and its associated proteoforms can form fibrillar aggregates that contribute to neurodegeneration through the mediation of oxidative stress, mitochondrial dysfunction, and synaptic loss [2]. Although a large body of research in the past two decades has been focused on two major proteoforms of Aβ, namely Aβ_40_ and Aβ_42_, ample evidence suggesting the presence of several shorter and post-translationally modified proteoforms in brains and CSF of Alzheimer’s disease patients has accumulated in the last two decades [3, 4]. These proteoforms vary in their seeding proficiencies, three-dimensional conformations, transport mechanisms, and toxicity [5–8]. Together with tau tangles, Aβ plaques constitute the major hallmarks of Alzheimer’s disease.

Alzheimer’s disease is the most common form of dementia and affects approximately 10% of the population above 65 years of age [9]. Clinically, it is defined as memory impairment accompanied by changes in executive function, visuospatial capability, speech, behavior, and movement. The recently discovered rapidly progressive variant of Alzheimer’s disease (rpAD), frequently misdiagnosed as Creutzfeldt-Jakob disease, constitutes a small subset of Alzheimer’s disease patients. rpAD follows the pathophysiological course of typical AD (sAD) but presents a faster decline in cognition and shorter survival time [10]. No significant differences are evident among sAD and rpAD patients regarding brain atrophy, hippocampal volume, genetic determinants, molecular hallmarks, neurological symptoms, or CSF biomarkers (tau, phospho-tau, Aβ_40_, and Aβ_42_). However, rpAD cases show higher CSF concentrations of 14–3-3 protein and a significant lack of apolipoprotein E ε4 allele homozygosity in comparison to sAD [11].

The existence of multiple clinical variants of Alzheimer’s disease with seemingly similar underlying pathology and molecular players is a dilemma for the research community. The key to distinct behavior of Aβ and tau, which can affect the progression rate of Alzheimer’s disease, may lie in the strain theory of prion disorders. Like the Scrapie isoform cellular prion protein (PrP^Sc^), other amyloidogenic proteins also exist as varied strains that arise by slight modifications in molecular profiles and differ in transmission. The conformational characteristics of these strains are transmitted into the host, where they propagate, causing distinct phenotypes with the same underlying pathology [12–14]. Previously, Aβ proteoforms have been shown to not only possess distinct biochemical signature but also different stability, distribution, and morphology in the brain. Moreover, they are transmissible among humans and between humans and animals [15]. These variants fulfill the definition of strains and may underlie the clinical heterogeneity of Alzheimer’s disease cases.

The current study was designed to establish comprehensive molecular profiles of endogenous Aβ proteoforms, featuring their composition, quantities, biosynthesis, aggregation kinetics, structure, and interactions, and to determine their potential to give rise to various strains in Alzheimer’s disease cases presenting typical and rapid progression.

## Methods

### *Collection of Brain Samples*

All frontal cortex samples were obtained from the Institute of Neuropathology Brain bank, Barcelona (HUB-ICO-IDIBELL Biobank) in accordance with Spanish legislation (Ley de la Investigación Biomédica 2013 and Real DecretoBiobancos, 2014) following informed consent of participants or their legal next of kin and the approval of the local ethics committee. The study was approved by the local ethics committee in Göttingen (No. 24/8/12). All Alzheimer’s disease cases met the Consortium to Establish a Registry for Alzheimer’s Disease (CERAD) criteria while the rpAD patients fulfilled the current definitions of rpAD [16]. The preliminary probable diagnosis on our rpAD cohort was that of a prion disease, owing to a rapid memory decline; however, the neuropathological assessment indicated a confirmed diagnosis of AD. Samples with copathologies that may contribute to rapid decline and those with a family history of Alzheimer’s disease were excluded from this cohort. The non-demented controls were chosen to ensure that they had no underlying pathologies that might contribute towards neurodegeneration. The sample selection was aimed to ensure that no significant differences in postmortem delay were evident among various experimental groups. A summary of all the samples used for this study is presented in Table 1 and Supplementary Fig. 1.

**Table 1:** Clinical data of brain samples utilized in the current study. For all sAD, rpAD, and control cases, Aβ pathology was scored based on the CERAD scoring system, while the Braak and Braak staging system was used to score NFTs

No	Patient ID	Clinical diagnosis	Disease duration (years)	Gender	Age	Braak stages
1	Control 1	-	-	Male	86	II/A
2	Control 2	-	-	Male	69	II/A
3	Control 3	-	-	Male	68	I/0
4	Control 4	-	-	Male	77	I/A
5	Control 5	-	-	Male	67	I/0
6	Control 6	-	-	Female	73	I/0
7	Control 7	-	-	Male	61	I/0
8	Control 8	-	-	Male	74	II/A
9	sAD 1	AD	> 4	Female	56	V/C
10	sAD 2	AD	> 4	Female	85	V/C
11	sAD 3	AD	> 4	Female	81	V/C
12	sAD 4	AD	> 4	Female	81	IV/C
13	sAD 5	AD	> 4	Female	82	V/B
14	sAD 6	AD	> 4	Male	81	IV/B
15	sAD 7	AD	> 4	Male	82	V/C
16	sAD 8	AD	> 4	Male	66	V/C
17	sAD 10	AD	> 4	Female	79	I/A
18	sAD 11	AD	> 4	Female	79	I/A
19	sAD 12	AD	> 4	Female	86	II/A
20	sAD 13	AD	> 4	Male	83	III/0
21	sAD 14	AD	> 4	Female	71	III/0
22	sAD 15	AD	> 4	Male	64	II/A
23	rpAD 1	rpAD	< 4	Male	83	VI/C
24	rpAD 2	rpAD	< 4	Female	77	IV
25	rpAD 3	rpAD	< 4	Female	85	V
26	rpAD 4	rpAD	< 4	Female	85	IV
27	rpAD 5	rpAD	< 4	Male	83	VI/C
28	rpAD 6	rpAD	< 4	Male	65	-
29	rpAD 7	rpAD	< 4	Female	86	-
30	rpAD 8	rpAD	< 4	Female	75	III

### Preparation of Brain Extract

For immunoprecipitation, brain tissues (10% w/v) were homogenized in tris lysis buffer [50 mM Tris–Cl (pH 8.0), 0.5% CHAPS, 1% Triton X100, 1 mM DTT, protease, and phosphatase inhibitors], followed by overnight incubation at 4 °C. The samples were centrifuged at 14,000 rpm for 45 min and the resultant supernatant was saved as tris-soluble fraction. The pellet was resuspended in 70% formic acid via sonication and the resultant slurry was incubated at room temperature for 20 min. The supernatant from the subsequent centrifugation was saved as FA-soluble fraction. Proteins from Tris-soluble fraction were quantified by Bradford assay (Bio-Rad, USA) while those from FA-soluble fraction were quantified by measuring the absorbance at 280 nm using Nanodrop Spectrophotometer due to low quantity and incompatibility with the assay reagents (Thermo Fisher Scientific, USA).

For 1D PAGE, brain tissues (10% w/v) were homogenized in urea-thiourea lysis buffer (7 M urea, 2 M thiourea, 4% CHAPS, 1% DTT, protease, and phosphatase inhibitors), followed by overnight incubation at 4 °C. The samples were centrifuged, and the supernatant was utilized for analysis after quantification using Bradford assay (Bio-Rad, USA).

### Immunoprecipitation (IP)

IP of Aβ was performed as previously described [17]. Briefly, 4 ul of two Aβ antibodies, 4G8 and 6E10 (Biolegend, USA), were coated on magnetic beads (Dynabeads; Invitrogen, USA). Dynabeads (1.5 mg/0.5 mg of protein sample) were given two washes with 0.3% CHAPS and incubated with 4 μl each of two Aβ antibodies, 4G8 and 6E10, for 30 min at 4 °C. Tris-soluble fraction, 500 ug protein, was added to the coated beads directly while the FA-soluble fraction was neutralized with 5 M NaOH in 1 M Tris prior to addition, and then the mixture was incubated overnight at 4 °C. Subsequently, the beads were washed with 0.3% CHAPS to remove non-specific proteins bound to the beads. The samples were either eluted in rehydration buffer (8.3 M urea, 0.5% CHAPS, and 20 mM DTT) for 2D-PAGE or 10% formic acid for top-down mass spectrometry by rotating the beads at room temperature for 10 min.

### Polyacrylamide Gel Electrophoresis and Immunoblot Analysis (PAGE-IB)

Isoelectric focusing was performed with minor modifications in the protocol described previously with pH 3–10, 7 cm, non-linear IPG strips [[18], Bio-Rad, Germany]. The proteins were further resolved using 4–12% gradient Bis–Tris gels (Thermo Fischer Scientific, USA) and transferred onto 0.20-µm PVDF membranes under semidry conditions (1 mA/cm^2^, 45 min), followed by incubation in boiling PBS for 3 min for antigen retrieval. The membranes were blocked and incubated in 6E10 antibody (1:1000) overnight. They were rinsed with PBS-T followed by incubation in horseradish peroxidase-conjugated secondary anti-mouse antibody (1:10,000) for 1 h. The chemiluminescent signal was detected using an enhanced chemiluminescence solution and ChemiDoc™ Imaging System (Bio-Rad, Germany). The pI (Isoelectric point) computational tool from Expasy was used to generate a reference table for pIs of different proteoforms (Expasy, SIB Swiss Institute of Bioinformatics). The images were analyzed using Delta 2D software (Version 4.8, Decodon GmbH, Germany).

The semi-quantitative analysis of various Aβ cleaving enzymes, APP, and Aβ_Total_ was performed using 1D SDS-PAGE. Immunoblotting was performed as described in the previous section. The expressions of α-secretase, β-secretase, γ-secretase, PSEN-2, Nicastrin, IDE, plasminogen, APP (6E10), and Aβ_Total_ (6E10) were assessed. All blots were stained with MemCode™ reversible protein stain according to the manufacturer’s instructions and normalized through total protein normalization The images were analyzed using Image Lab software (Version 6.0, Bio-Rad, Germany). Data from three independent experiments were used for statistical analysis. Information about the antibodies used in this study has been summarized in Supplementary Table 1.

### Matrix-Assisted Laser Desorption/Ionization Mass Spectrometry (MALDI-MS)

Matrix was prepared by dissolving 10 mg of sinapinic acid (Sigma, Germany) in 1.0 ml of 50% acetonitrile, 50% proteomics grade water, and 0.1% trifluoroacetic acid (TFA). Dried IP eluates were resuspended in 0.1% TFA and mixed with the matrix in a ratio of 1:1. 1.5 µl of each sample was deposited on the MALDI plate and allowed to cocrystallize at room temperature. Spectra were calibrated using readymade peptide calibration standard (Bruker Daltonics, USA). Peaks were acquired using repiflex MALDI Tissuetyper (Bruker Daltonics, USA) in a m/z range of 2000 to 6000 using positive linear mode. Five measurements were taken for each sample and the average spectrum was generated. Peaks were analyzed in FlexAnalysis (Version 3.4, Bruker Daltonics, USA) and Aβ proteoforms were manually annotated based on m/z values. Proteoforms with a signal/noise ratio ≥ 2 and deviation of no more than 5 Da from theoretical mass were included in the analysis. Proteoforms that were detected in at least two out of three independent replicates were included in the final dataset.

#### ELISA

N and C-terminally truncated proteoforms of Aβ were quantified using Aβ_x-42_ (Biolegend, Germany), Aβ_1-x_ (IBL International, Germany), and Aβ_1-40_ (Biosource, USA) ELISA kits according to the manufacturer’s instructions. Supplementary Table 1 contains detailed information about the kits.

### Real-Time Quaking Induced Conversion (RT-QuIC)

Amyloid fibrils were extracted using minor modifications in the previously optimized protocol from 100 mg of brain tissue [19]. Purified fibrils were resuspended in 15 µl of RT-QuIC seeding buffer and quantified by Nanodrop spectrophotometer. Half of the brain extract (7.5 µl; 2–3 µg/µl) was further diluted with the seeding buffer to a final volume of 88 µl and sonicated on ice for 10 min. Synthetic Aβ_40_ and Aβ_42_ (Abcam, UK) were diluted in DMSO (50 µM) and sonicated for 30 min immediately prior to the reaction and added to diluted brain extract along with 2 µl of Thioflavin-T in PBS (Th-T; 1 mM) solution. The final reaction volume of each mixture was 100 µl. Multiple technical replicates from each sample were incubated simultaneously in FLUOstar Omega plate reader for 46 h at an intermittent shaking mode (600 rpm for 1 min after every 29 min) at 37 °C. Fluorescent measurements were recorded every 30 min (excitation 450 nm, emission 480 nm) and used for analysis.

### Confocal Laser Scanning and Atomic Force Microscopy (AFM)

Preliminary analysis for the structure of brain-derived fibrils was conducted using confocal laser scanning microscopy. Th-T dye (1 mM) was added to RT-QuIC products in a ratio of 1:10. The resulting mixture (1 µl) was added to glass slides and imaged immediately at 488 nm using Zeiss LSM 510 Meta Confocal laser scanning microscope.

For AFM, RT-QuIC products (5 µl) were added to freshly stripped micas and incubated for 20 min at room temperature. The coated micas were washed three times with ultrapure H_2_O (10 µl) to remove salts and other impurities and excess H_2_O was removed with a gentle nitrogen stream. The samples were imaged in intermittent contact mode (tapping mode) in MFP-3D Infinity microscope using Olympus microcantilevers (OMCL-AC160TS) at a drive frequency of 260.058 kHz, guided by Igor Pro software. The scan area for each image was 10 µm^2^ and the scan rate was 0.5 Hz. Analysis of the acquired images was performed via the open-source software Gwyddion.

### Toxicity Assays

SH-SY5Y cells (30,000 cells/well) were plated in a 96-well plate in Optimem (Gibco, Germany) supplemented with 1% penicillin/streptomycin at 37 °C, 5% CO_2_. After 24 h, the medium was replaced with 100 ul Optimem containing RT-QuIC products (20 µM), and then incubated for another 24 h. MTS reagent (10 µl) was added to each well and the absorbance 490 nm was recorded after 3 h.

### Liquid Chromatography/Electrospray Ionization Tandem Mass Spectrometry (LC-ESI MS/MS)

Interacting partners of Aβ in tris-soluble fraction were identified via co-immunoprecipitation. The proteins that copurified with fibrils, on the other hand, were identified directly. The targeted proteins were digested with trypsin overnight at 37 °C. The peptide mixtures were concentrated on a reversed-phase C18 precolumn and separated on a reversed-phase C18 nanoflow chromatography column (self-packed with Reprosil-Pur C18 AQ 3 µm material) using a linear gradient (5–35% acetonitrile vs. 0.1% FA; 15 min) at a flow rate of 300 nL/min in an Easy nLC-1000 nanoflow chromatography system. Q Exactive hybrid quadrupole/orbitrap MS system (paired with Excalibur software) was used to analyze the eluates using Top10 method in the data-dependent acquisition mode. Tandem mass spectra were obtained using Raw2MSM software. MS/MS spectra were analyzed using Mascot instructed for searching Swissprot Homo sapiens reference proteome (revision 10.2018) with a mass tolerance of 10 ppm for precursors and 0.05 Da for fragments. Methionine oxidation was regarded as a variable PTM whereas cysteine modification was set as a fixed modification. MS/MS-based identification was validated using Scaffold software. A confidence threshold greater than 95.0% was used for accepting peptide identifications while a confidence threshold of 99.0%, paired with a minimum of two identified peptides, was employed as a prerequisite for accepting protein identification.

### Statistical Analysis

The data were analyzed and visualized using PRISM and RStudio. *P*-values were determined using either one-way ANOVA followed by Tuckey’s post hoc test or unpaired Student’s *t*-test; values ≤ 0.05 were considered significant. All data are expressed as mean ± standard error of the mean (SEM) unless stated otherwise.

## Results

### Purification of Endogenous Aβ Proteoforms from sAD and rpAD Brains

We divided the brain proteome into two pathologically relevant fractions, namely the tris and the formic acid-soluble (FA-soluble) fractions. Tris-soluble fractions comprise a smaller, soluble Aβ species that impart toxic effects within the cell body. The FA-soluble fraction, on the other hand, corresponds to insoluble Aβ species deposited as fibrils and plaques that sequester circulating Aβ and may function as a reservoir [20, 21]. Aβ-enriched fractions were prepared by hybrid IP, and the capture of various Aβ proteoforms was validated using 2D-PAGE.

**Fig. 1:**
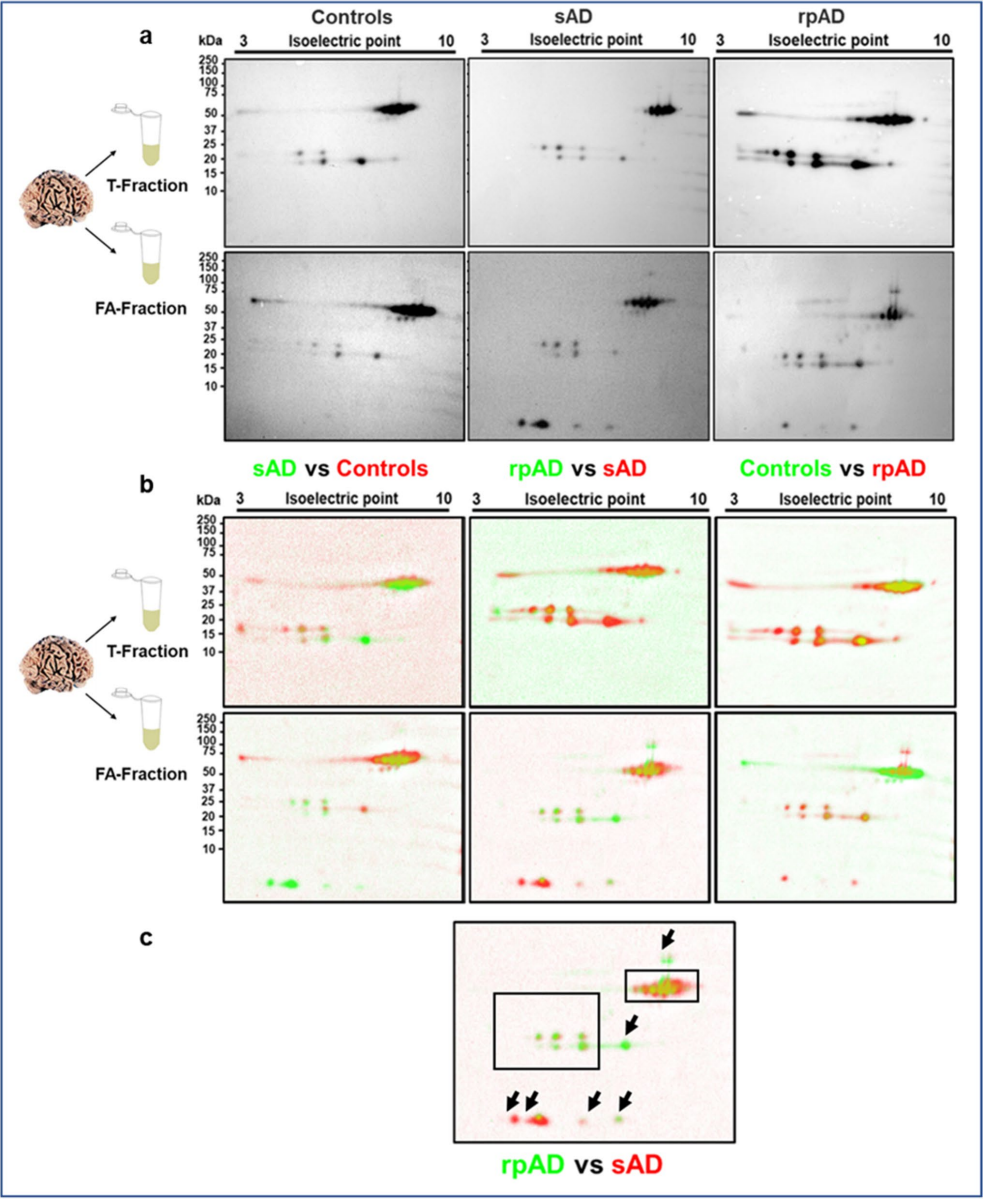
Validation of monomeric and oligomeric Aβ proteoforms in enriched extracts from sAD, rpAD, and control cases. (**a**) 2D Western blot indicated various N and C-terminally truncated Aβ proteoforms as monomers, pentamers, hexamers, and dodecamers in Tris and FA-soluble fractions isolated from brain. (**b**) Delta 2D visualized the presence of differentially expressed Aβ proteoforms in sAD, rpAD, and control cases. (**c**) Arrowheads indicate the differentially expressed proteoforms among AD and rpAD cases. T-fraction stands for the tris-soluble fraction

Bands for Aβ monomers and oligomers were obtained at 4 kDa, 20 kDa, 24 kDa, and 56 kDa, corresponding to monomers, pentamers, hexamers, and dodecamers, respectively. The pI-based pattern was in accordance with the previous reports [18, 22–25]. Although intersubject variability in expression was evident throughout the cohort, all tested sAD FA-soluble fractions cases showed spots at pI of 5.31 (corresponding to C-terminally truncated proteoforms, including Aβ_40_, Aβ_42_, Aβ_38_), 5.76 (presenting shorter N and C-terminally truncated proteoforms including Aβ_20_, Aβ_2-14_, Aβ_3-14_), and 6.27 (showing N-terminally truncated proteoforms including Aβ_4-42_). An additional spot was detected at pI of 4.89 for one sAD case, indicating the presence of intermediate C-terminally truncated proteoforms including Aβ_26_. In rpAD cases, the two major spots detected were at 5.31 and 6.27. Only one case showed a faint spot at 5.71. The presence of spots at pI other than 5.31 validated the presence of smaller, less common proteoforms in addition to Aβ_40_ and Aβ_42_ in our enriched samples and predicted differences between their signatures in sAD and rpAD (Fig. 1). The amount of monomeric Aβ in other experimental groups was too low to be detected.

### *Aβ*_*40*_*, Aβ*_*42*_*, Aβ*_*4-42*_*, Aβ*_*11-42*_***, and Their Pyroglutamate Counterparts Are the Major Proteoforms in FA-Soluble Fraction***

As predicted by 2D-PAGE-IB, MALDI-MS was able to obtain identifiable peaks of monomeric Aβ proteoforms only in FA-soluble fractions of sAD and rpAD brains. Aβ_40_ was detected in FA-soluble fractions of some control cases. However, we excluded these cases from the analysis, as the quality of peaks was poor, resulting in non-reproducible findings. The tris-soluble fractions presented a pattern similar to our negative controls, indicating that the amount of Aβ was below the detection limit (Supplementary Fig. 2).

**Fig. 2:**
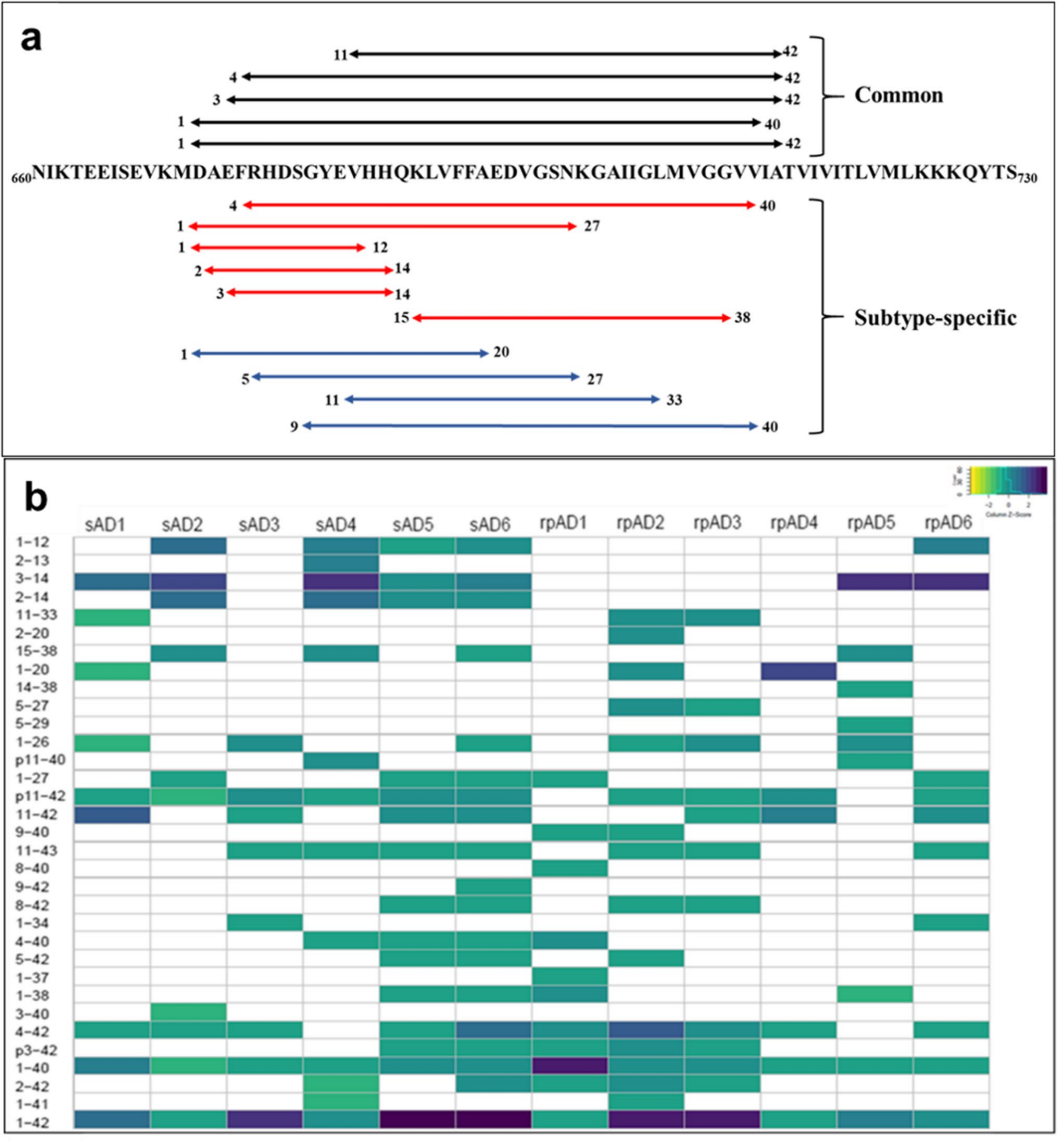
Diversity in proteoforms identified in formic acid-soluble fraction of sAD and rpAD. Top-down MALDI-MS identified 33 different proteoforms of Aβ. Although intersubject variability is evident in proteoform signature obtained from various cases, Aβ_p3-42_, Aβ_3-42_ Aβ_p11-42_, Aβ_11-42_, Aβ_4-42_, Aβ_1-40_, and Aβ_1-42_ were the most dominant proteoforms. (**a**) The sequence of proteoforms common in sAD (red), rpAD (blue), or both groups (black) is marked on APP (only sequence between amino acid 660 to 730 is shown). (**b**) The heatmap depicts the relative intensities of all identified proteoforms, calculated using the average area under the curve (AUC) from five measurements. The intensities were normalized for each sample and the respective *Z*-scores of proteoforms were used for this plot

Among the 33 proteoforms included in the final dataset, Aβ_40_, Aβ_42_, Aβ_4-42_, Aβ_11-42_, Aβ_3-42_, pyroglutamate Aβ_11-42_ (Aβ_p11-42_), and pyroglutamate Aβ_3-42_ (Aβ_p3-42_) were common to both sAD and rpAD cases. Aβ_42_ was the most abundant proteoform in all cases studied, other than one rpAD sample. Aβ_1-12_, Aβ_2-14_, Aβ_3-14_, Aβ_15-38_, and Aβ_4-40_ were found to be more common in sAD cases, whereas Aβ_5-27_, and Aβ_9-40_ were more common in rpAD cases (Fig. 2a). A heatmap depicting the relative amounts of various proteoforms extracted from individual cases is presented in Fig. 2b.

### The Expression of APP, Aβ Proteoforms, and Aβ-Processing Enzymes is Highly Similar in sAD and rpAD

**Fig. 3:**
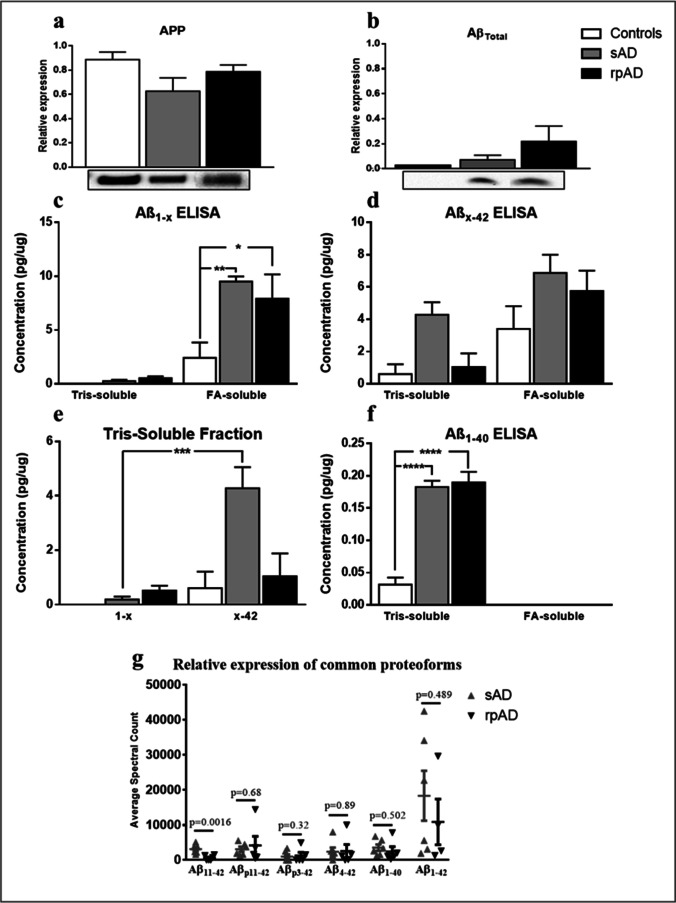
Relative expression of APP and various proteoforms of Aβ in brain samples. The expression of (**a**) APP, (**b**) Aβ_Total_, (**c**) C-terminally truncated proteoforms, (**d**) N-terminally truncated proteoforms, and (**f**) Aβ_40_ in controls, sAD and rpAD cases quantified using IB and ELISA analysis (*n* = 4–6). (**e**) Comparison of various truncations within the tris-soluble fraction is also presented. (**g**) The average spectrum counts obtained for common proteoforms, other than Aβ_11-42_, by MALDI-MS also reflected results similar to ELISA analysis. All blots were subjected to total protein normalization and data from three independent experiments was used for densitometric analysis while the ELISA averages were obtained by analyzing all samples as duplicates. Error bars represent SEM. One-way ANOVA, followed by Tukey’s multiple comparisons test, was used for statistical analysis. (*p ≤ 0.05; **p ≤ 0.01; ***p ≤ 0.001)

**Fig. 4:**
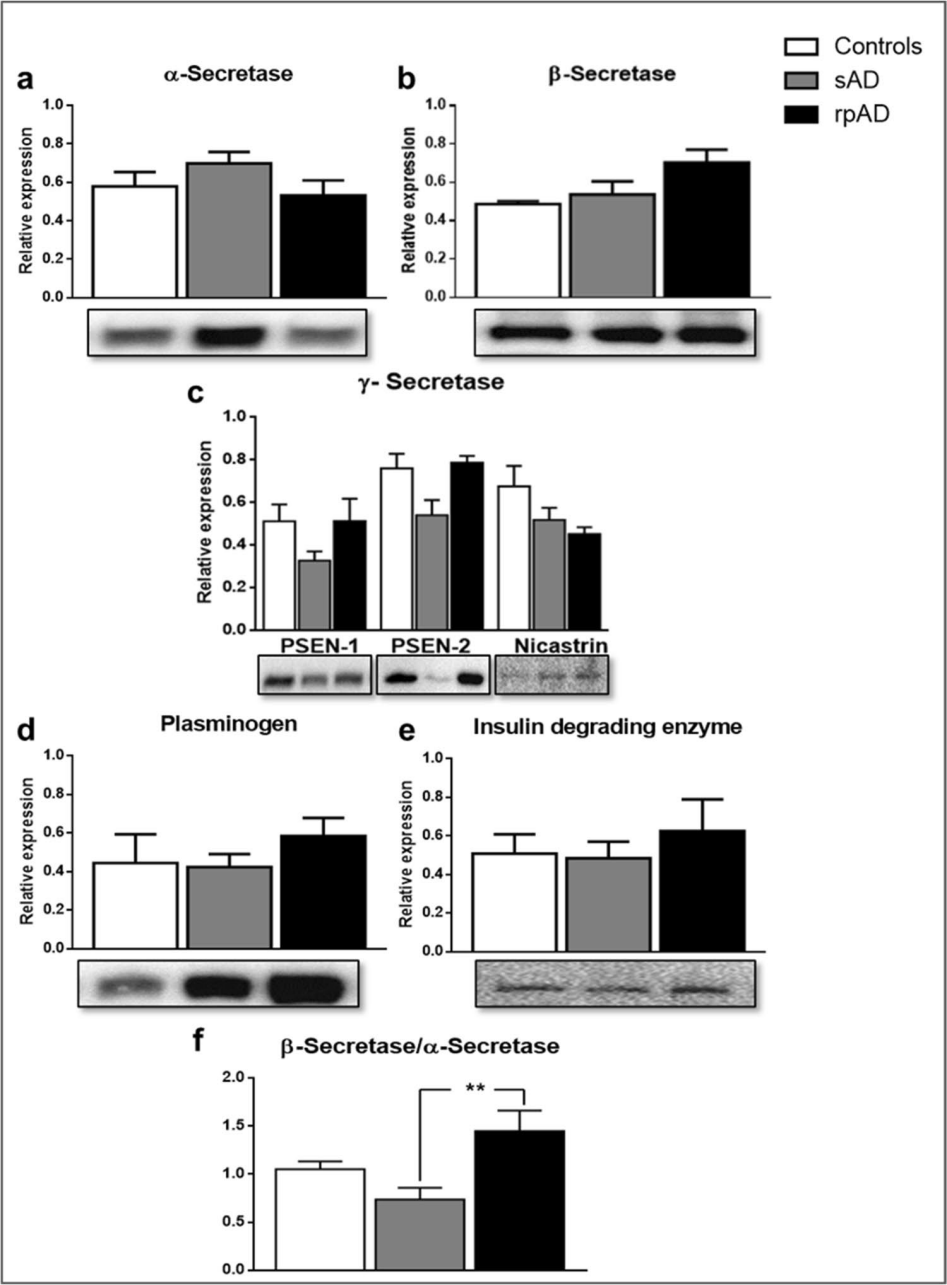
Western blot analysis for the relative expression of major Aβ cleaving enzymes. The relative expression of (**a**) α-secretase, (**b**) β-secretase, (**c**) γ-secretase [Presenilin-1 (PSEN-1); Presenilin-2 (PSEN-2); Nicastrin], (**d**) Plasmin, and (**e**) Insulin degrading enzyme (IDE) in non-demented controls, sAD and rpAD cases depicted no significant differences (*n* = 6–12). (**f**) However, the higher expression of β-secretase in comparison to α-secretase indicated differences in amyloidogenic processing between the two variants. All blots were subjected to total protein normalization. Densitometric analysis was conducted using data from three independent experiments. One-way ANOVA, followed by Tukey’s multiple comparisons test, was used for statistical analysis. Error bars represent SEM. (**p ≤ 0.01)

Aβ proteoforms, their precursor APP and Aβ cleaving enzymes were quantified using the data obtained from MALDI-MS dataset in addition to ELISA and IB analysis. However, no significant differences were evident for either target among the sAD and rpAD experimental groups (Fig. 3 and Fig. 4). In comparison to controls, the amount of C-terminally truncated proteoforms was significantly higher in FA-soluble fraction of sAD and rpAD cases. Similarly, the ELISA for N-terminally truncated proteoforms also showed the largest amounts in FA-soluble fraction of sAD, followed by rpAD and controls, but the differences were not significant. However, no such trends were evident in the tris-soluble fraction in either ELISA (Fig. 3d and e).

In tris-soluble fractions, ELISA results showed a lower amount of C-terminally truncated proteoforms in comparison to N-terminal truncations in all control, sAD, and rpAD cases. This trend was especially evident in sAD cases, where the amount of N-terminally truncated Aβ was significantly higher than its C-terminal counterparts, possibly because these shorter proteoforms are less prone to aggregation and are frequently formed during the clearance of larger, highly aggregated proteoforms (Fig. 3e). FA-soluble fractions, on the other hand, showed no significant differences between N and C-terminally truncated pools. Aβ_40_ was only detectable in tris-soluble fractions in our ELISA experiments and was also not significantly different between sAD and rpAD cases (Fig. 3f). Similarly, the quantitative data from MALDI-MS experiments only detected significant differences in the relative quantity of Aβ_11-42_ among all the proteoforms common to both clinical variants of Alzheimer’s disease (Fig. 3g).

We did not observe any significant differences in the expression of enzymes that take part in generation and clearance of Aβ either (Fig. 4a-e). However, the expression of beta-site amyloid precursor protein cleaving enzyme 1 (BACE1), our targeted β-secretase, relative to α-secretase (A disintegrin and metalloproteinase domain-containing protein 10; ADAM10), was significantly higher in rpAD in comparison to other groups, indicating increased cleavage of Aβ through the amyloidogenic pathway in these cases (Fig. 4f).

### Brain-Derived Aβ from sAD and rpAD Cases Features Different Aggregation Kinetics and Varies in Size and Morphology

**Fig. 5:**
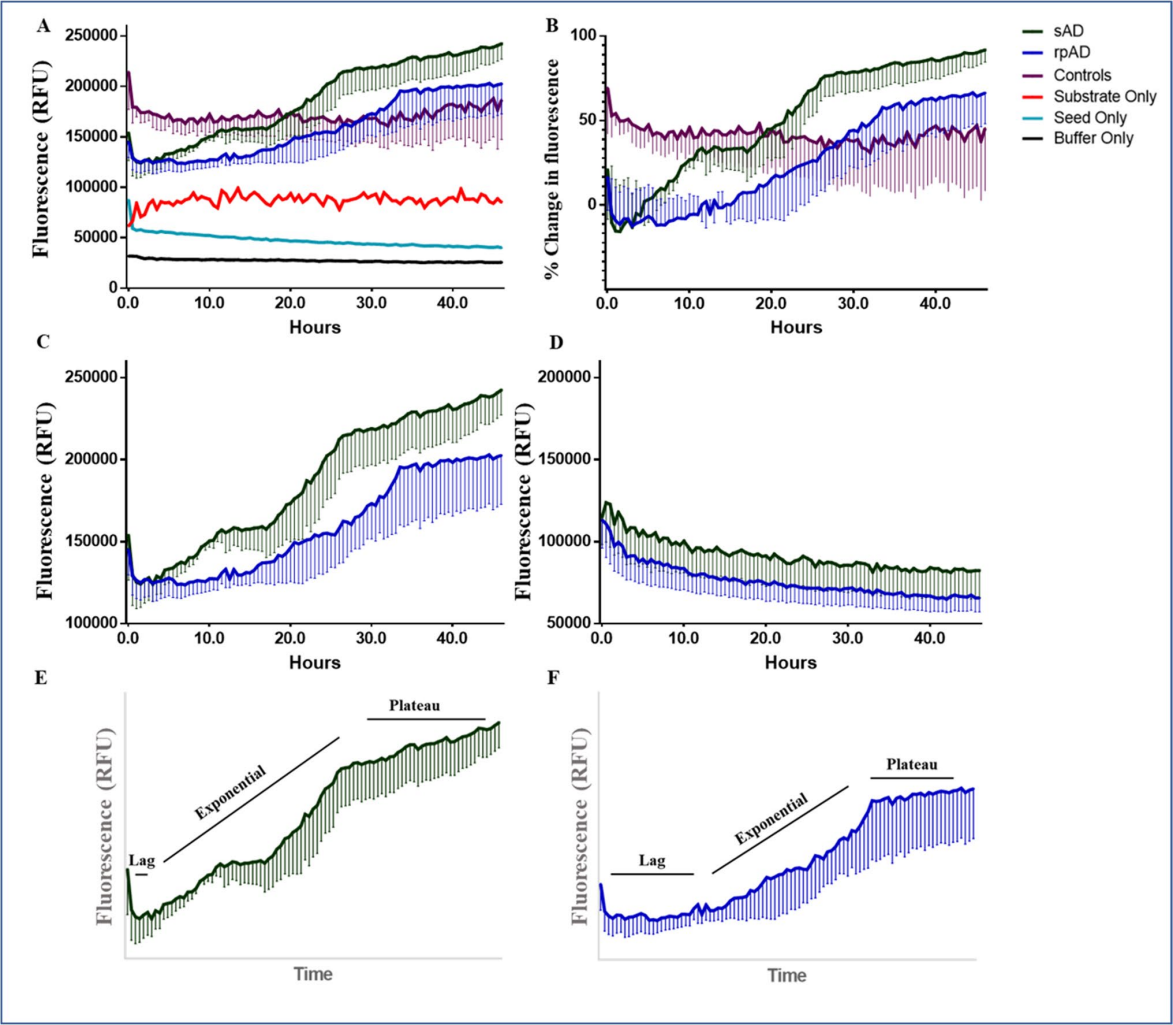
Kinetic curves obtained for Aβ RT-QuIC reactions seeded with the fibrillar extract from sAD, rpAD, and controls. (**a**) The kinetic curves plotted using an average of four measurements recorded for each of three biological replicates in every experimental group (**b**) Non-demented controls depicted higher absorbance in comparison to seed-only and substrate-only controls. However, no increase was recorded in the signal throughout the experiments, indicating that no seeding occurred in this group. (**c**) Only the rpAD and sAD showed an increase in Th-T signal and seeding occurred faster in sAD cases. (**d**) Seeds extracted in PBS (corresponding to tris-soluble fraction) did not undergo aggregation under these reaction conditions. The relative duration of lag and exponential phase for (**e**) sAD and (**f**) rpAD cases is also presented. Error bars represent SEM

As our IB and ELISA experiments detected no significant differences in the quantities of common Aβ proteoforms, we next targeted the differences in aggregation and structures of brain-derived Aβ. Seeds purified via ultracentrifugation were amplified in the presence of synthetic Aβ_40_ and Aβ_42_ as substrate, and their subsequent RT-QuIC profiles were utilized to establish differences in their aggregation kinetics. The complications caused by self-aggregation of the substrate and variable amounts of seeds were resolved before hand (Supplementary Fig. 3). The samples without any substrates (seed-only controls) were not positive for RT-QuIC (Fig. 5). A similar trend was also observed in controls, where the signal showed no increase throughout the reaction, indicating that Aβ proteoforms in these cases were probably not enough to seed the conversion under our reaction conditions (Fig. 5b). Only reactions seeded with the extract from sAD and rpAD brain showed an increase in Th-T signal in this experiment. Interestingly, the conversion of monomeric substrate to its fibrillar, β-sheet rich counterpart was faster in sAD cases in comparison to rpAD, as indicated by kinetic curves in Fig. 5c. Seeds corresponding to tris-soluble fraction, on the other hand, showed no such trend (Fig. 5d).

**Fig. 6:**
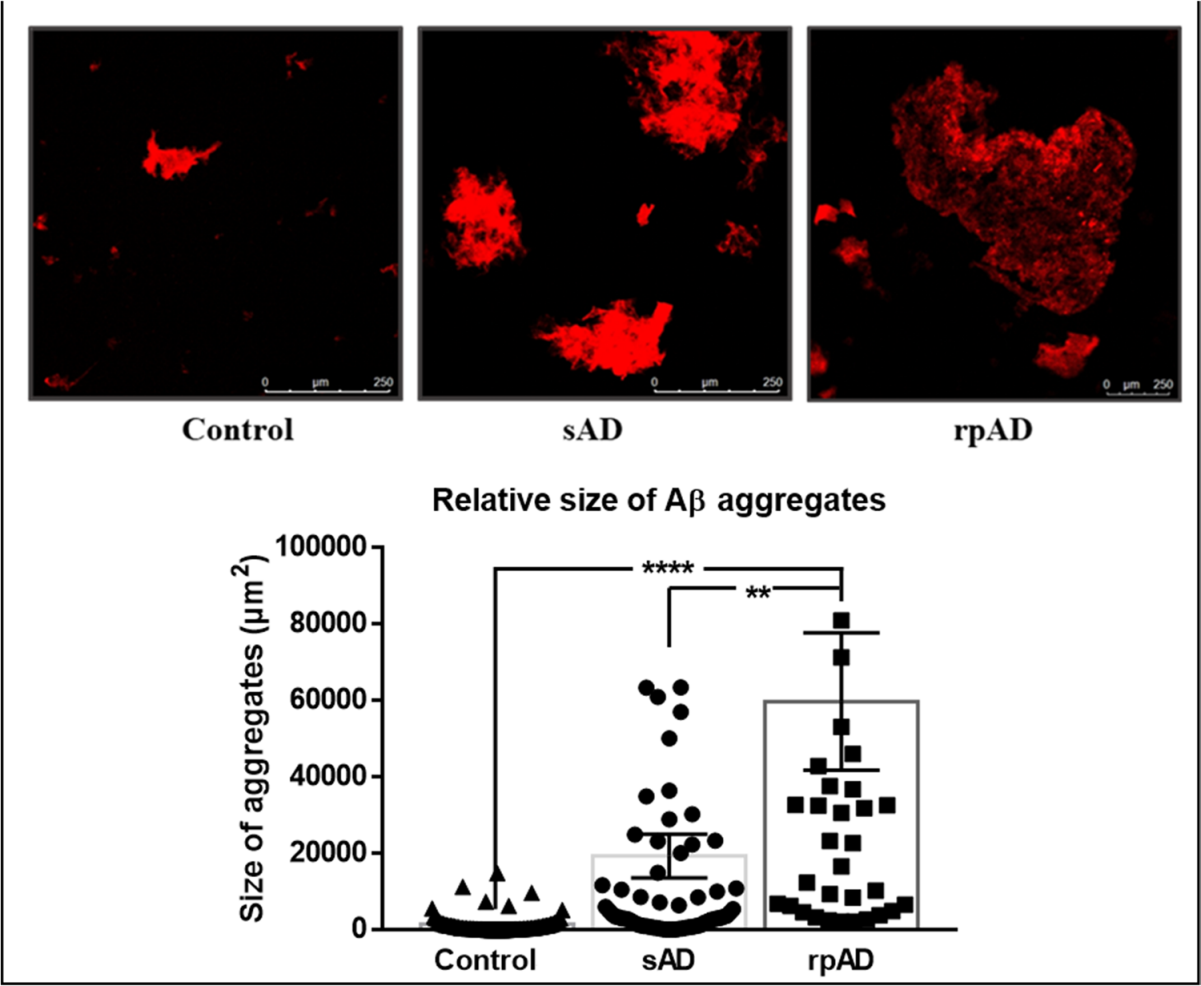
Differences in size of aggregates from sAD and rpAD visualized by Th-T staining and confocal microscopy: The size of the aggregates was calculated by measuring the average size of 40–50 structures per experimental group (*n* = 3). The aggregates from rpAD were significantly larger than those observed for controls and sAD. Dots represent individual data points. One-way ANOVA, followed by Tukey’s multiple comparisons test, was used for statistical analysis. Scale bar represents 250 µm and error bars present SEM (**p ≤ 0.01; ****p ≤ 0.0001)

Differences in Aβ aggregates seeded using extracts from sAD, rpAD, and control brains were visualized using confocal and atomic force microscopy. The RT-QuIC reactions seeded with rpAD brain extracts yielded significantly larger aggregates, in comparison to aggregates generated by sAD and non-demented controls, despite their slower rates of aggregation. sAD cases, on the other hand, produced smaller but more frequent aggregates, but their size was not significantly different from controls (Fig. 6). Smaller aggregates were also present in seed and substrate only; however, their frequency was too low for analysis, so these samples were not included in the graph.

**Fig. 7:**
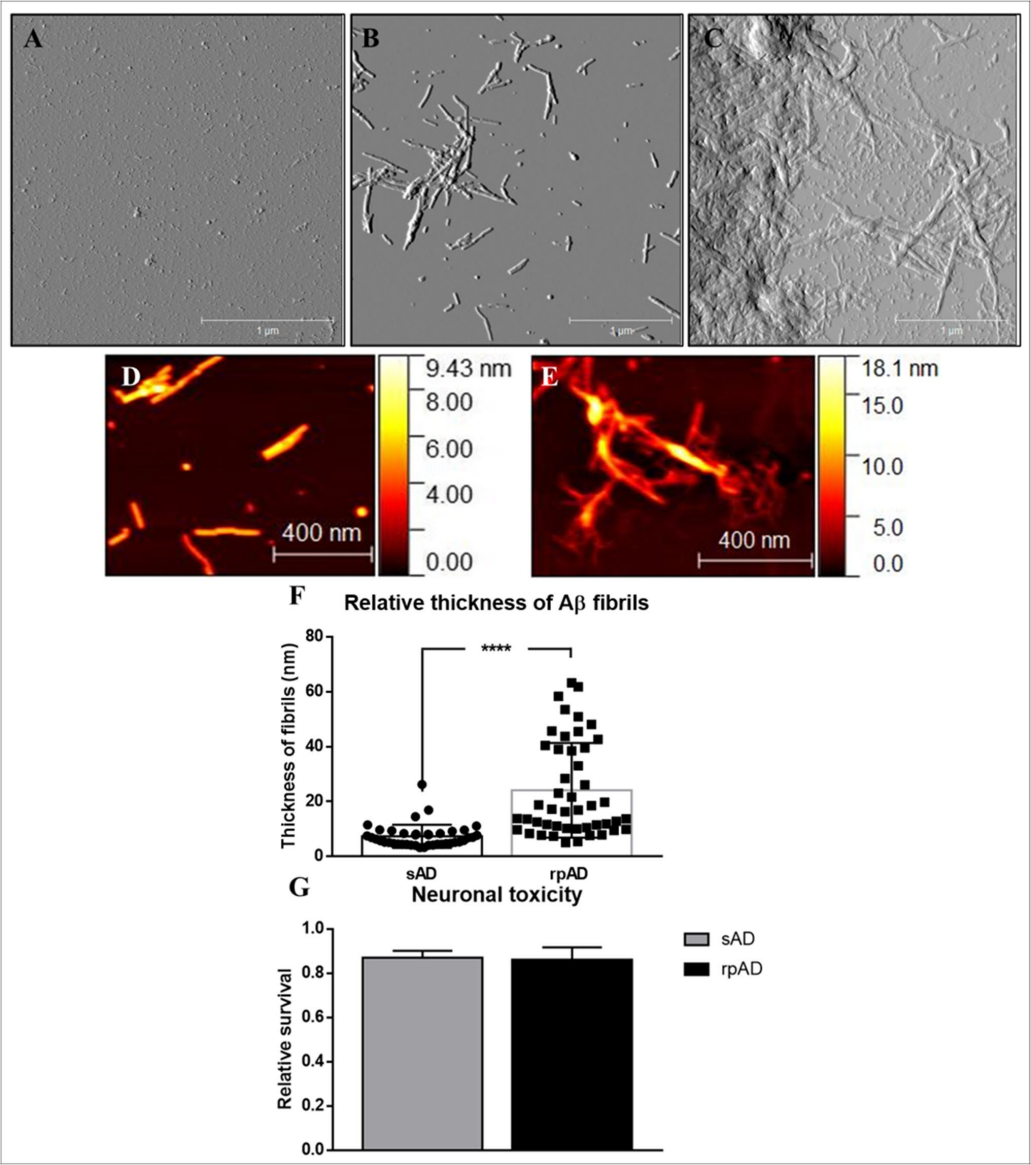
Amplitude and height-retrace images obtained for RT-QuIC products via tapping mode atomic force microscopy: (**a**) Control cases presented globular seeds only whereas sAD and rpAD cases (**b** and **c**) showed fibrillar and amorphous aggregates as observed in respective amplitude images. Magnified figures present the detailed structure and the thickness of fibrils from sAD (**d**) and rpAD (**e**) brains. (**f**) rpAD cases featured thicker fibrils in comparison to sAD cases. The height scale presents the approximate thickness of individual fibrils. The height of fibrils was calculated by measuring the average size of 20–30 fibrils per experimental group (*n* = 3). Scale bar represents 1 µm and 400 nm. Dots represent individual data points. (**g**) However, their toxicity in cells, relative to vehicle-treated cells, showed no significant differences among the two clinical variants of AD (*n* = 3) when applied to SH-SY5Y cells. The graph shows an average of data acquired from three independent experiments. Statistical analysis was performed using unpaired Student’s *T*-test. Error bars represent SEM. (****p ≤ 0.001)

A similar trend was observed for the three experimental groups by using atomic force microscopy as well. rpAD samples featured large amorphous structures whereas sAD cases presented smaller aggregates with well-defined fibrils. The amorphous structures observed for rpAD may be products of highly hydrophobic fibrils that have higher propensity to bind with each other and generate a plaque-like morphology. Control samples just presented small globular structures verifying that these cases did not seed the aggregation of the substrate (Fig. 7).

The average thickness of fibrils generated by sAD and rpAD samples was calculated to further validate the differences in 3D folding of Aβ. The fibrillar structures observed in sAD seeded reactions had significantly lesser thickness in comparison to those seeded by rpAD extract (Fig. 7f). Importantly, only the thickness of distinct fibrils was measured and larger aggregates, where fibrils were buried inside the structure, were ignored to avoid bias in data. Since no distinct fibrils were visible in control cases, their measurements were not included in the data set. However, the globular aggregates they formed had an average diameter of 200 ± 16.6 nm (mean ± SEM) and might just present seeds that did not undergo any aggregation.

### sAD and rpAD Derived Aβ Fibrils Are Equally Toxic in Their Final Conformations

The toxicity of Aβ proteoforms and their respective fibrillar aggregates relies on their ability to interact with various cellular components and organic molecules. The translation of the aforementioned differences in sAD and rpAD fibrils into functional relevance was therefore performed by investigating the relative toxicities in these fibrils in SH-SY5Y cells and flushing out their respective interacting partners from various fractions of diseased brains using co-IP and mass spectrometry (MS).

SH-SY5Y cells were treated with RT-QuIC products for 24 h and the toxicity was measured using MTS assay. Fibrils obtained from sAD and rpAD brains were more toxic to the cells than the vehicle-treated group. However, the differences within these two groups were not significant (Fig. 7g). Other experimental groups were intentionally removed from the dataset as the Aβ substrate in these cases did not undergo fibrillization in the time frame allowed and existed as the more toxic monomeric conformers. Their effect on the cells may misrepresent these cases as more toxic than sAD and rpAD fibrils are amplified.

### Distinct Partners of Aβ in sAD and rpAD

All proteins forming complexes with Aβ isolated from sAD, rpAD, and control brains were identified using co-IP followed by LC-ESI MS/MS. A total of 41 interactors were filtered and included in the final dataset after removing the common contaminants and the proteins that were reported in negative controls. Only the proteins that were reported in at least two out of four biological replicates for each experimental group with a spectrum count of more than 2 and a confidence threshold of 99.0% were included in the final dataset. The disease-specific distribution of Aβ interactors and the detailed characteristics of identified interactors are summarized in Table 2.

**Table 2:** Aβ interactors isolated from tris-soluble fractions of controls, sAD and rpAD. The list of Aβ interacting partners obtained through co-IP, along with their localization, function, and distribution, is summarized (*n* = 4). The localization and biological functions of identified Aβ interactors were annotated using the UniProtKB database. “A” stands or sAD, “R” for rpAD, “C” for controls, “Mit” for mitochondria, “Nu” for nucleus, “Cy” for cytoplasm, “Cysk” for cytoskeleton, “Mem” for cell membrane, and “ER” for endoplasmic reticulum

Identified proteins	UniProt ID	Localization	Functional category	Specificity
ATP synthase subunit beta	P06576	Mit	Energy metabolism	C, R
40S/60S ribosomal proteins	P62277	Nu	Translation	C, R
Actin-related protein 2, 3	P61160	Nu, Cysk	Cysk organization/Axon growth	C, R
Adenosylhomocysteinase 2	O43865	ER	Translation	C
Adenylate kinase isoenzyme	P00568	Cy	Energy metabolism	R
ADP/ATP translocase 1, 2	P12235	Mit	Energy metabolism	C, R
Band 4.1-like protein 3	Q9Y2J2	Mem, Cysk	Apoptosis, Cysk organization	C
Citrate synthase	O75390	Mit	Glucose metabolism	C, R, A
Cleavage and polyadenylation specificity factor	O43809	Nu	mRNA processing	R
Cysteine-rich protein 2	P52943	Cy	Cell division	C
Dihydropteridine reductase	P09417	Mit, Cy	Redox homeostasis	R, A
Dihydropyrimidinase-related protein 2	Q16555	Cy	Axon guidance	R, C
Fructose-bisphosphate aldolase C	P09972	Cy	Carbohydrate metabolism	All
GABA receptor-associated protein-like 2	P60520	Golgi	Transport	C, R
GTPase KRas	P01116	Cy	Signal transduction	C, R
GTP-binding nuclear protein Ran	P62826	Nu, Cy	Transport	All
Immunoglobulin superfamily member 8	Q969P0	Mem	Neurite outgrowth	C
LanC-like protein 1	O43813	Mem	Signaling	C, R
Microtubule-associated protein 1A	P78559	Cysk	Cysk organization, Axonal transport	All
Peptidyl-prolyl cis–trans isomerase A	P62937	Golgi	Protein refolding	A, R
Peroxiredoxin-2	P32119	Cy	Redox homeostasis	R
Phosphoglycerate kinase 1	P00558	Cy	Carbohydrate metabolism	All
Quinone oxidoreductase	Q08257	Cy	mRNA processing	R
Serine/threonine-protein phosphatase PGAM5	Q96HS1	Mit	Necrosis	R
Synaptotagmin-1	P21579	Mem	Neurotransmission	C, R
Tubulin beta-3 chain	Q13509	Cysk	Axon maintenance	C, R
Voltage-gated potassium channel subunit beta-2	Q13303	Mem	Neurotransmission	All

Additionally, in a separate experiment, the proteins that copurified with Aβ fibrils (seeds for Aβ RT-QuIC) and have the potential to function as accessory proteins for Aβ and alter the aggregation kinetics were also identified. All replicates from each experimental group were pooled to improve the quantity and detection of proteins. The dataset was then searched for targets that are amyloidogenic, promote amyloidogenesis, and prevent fibrillization of amylogenic proteins; the results are presented in Supplementary Table 2. Although the distribution was not very specific, sAD cases showed decreased levels of proteins that prevent fibrillization of Aβ in comparison to rpAD. Moreover, the concentration of amyloidogenic proteins that may potentiate fibrilization via cross seeding was also higher in sAD cases. These differences may underlie distinct aggregation kinetics of sAD and rpAD seeds in RT-QuIC reactions. Moreover, in vitro interactions between Aβ and neutralized proteins from FA-soluble fractions were also analyzed using co-IP experiments to validate the interactions of these accessory proteins. The potential of identified targets to interact with Aβ is summarized in Supplementary table 2.

## Discussion

Evidence from proteomic, biophysical, and animal studies has previously validated a distinct involvement of Aβ proteoforms in various subtypes of Alzheimer’s disease [26, 27]. More recently, the differences in the clinical presentation of Alzheimer’s disease cases have been attributed to the existence of Aβ strains [28]. Expanding on these findings, the present study established the presence of previously underrepresented Aβ proteoforms in sAD and rpAD brains, providing an insight into the biosynthesis and relative quantities of these proteoforms as well as evidence for the distinct 3D morphologies, interactions, and toxicities that may generate distinct strains and alter the course of Alzheimer’s disease.

In the current dataset, a total of 33 distinct proteoforms were identified, but Aβ_40_, Aβ_42_, Aβ_3-42_, Aβ_4-42_, Aβ_11-42_, Aβ_p3-42_, and Aβ_p11-42_ were the most abundant proteoforms in sAD and rpAD cases. Recent studies conducted on sAD brains also reported these targets as the most common Aβ proteoforms in the insoluble (FA-soluble) fraction of sAD brains [4, 29]. Unexpectedly, apart from one rpAD sample (rpAD1, Fig. 2), none of the samples presented subtype-specific differences in the signature of these major proteoforms. Pyroglutamate proteoforms were frequently detected in the plaque-associated proteome of both sAD and rpAD cases. Pyroglutamylation is known to increase the aggregation propensity of various proteoforms and the Alzheimer’s disease–associated behavioral deficits; hence, its presence indicates more toxic counterparts of Aβ proteoforms [30, 31]. In accordance with previously reported findings for sAD brains, N-terminally truncated Aβ was significantly higher than its C-terminal counterparts [22, 32].

Of the major proteoforms mentioned above, only Aβ_11-42_ had significantly different expressions in sAD and rpAD cases. However, despite its potential to aggregate aggressively, its toxicity is lower than the other known proteoforms, and the consequences of this difference remain to be understood [33]. Several other shorter proteoforms occurred more frequently in either sAD (Aβ_1-12_, Aβ_2-14_, Aβ_3-14_, Aβ_15-38_, and Aβ_4-40_) or rpAD (Aβ_5-27_ and Aβ_9-40_). The exact physiological and pathological roles of these subtype-specific proteoforms are also not yet known. In combination with a significantly higher ratio of β-secretase and potentially increased amyloidogenic processing of Aβ in rpAD cases, these results depict differences among the two clinical variants of Alzheimer’s disease at the post-translational stage of Aβ processing.

A lack of expressional differences in common Aβ proteoforms, especially the frequently targeted Aβ_40_ and Aβ_42_, prompted analysis of aggregation kinetics and structure. Based on the major proteoforms detected by MALDI-MS, a combination of Aβ_40_ and Aβ_42_ (in equal ratios) was used as a substrate for RT-QuIC reactions in contrast to using a higher concentration of either proteoform in an independent assay, which leads to self-aggregation. sAD cases were observed to aggregate faster with a shorter lag phase and steeper curves in comparison to rpAD. In a similar study with Aβ RT-QuIC, samples were divided into three groups depending upon the main Aβ proteoforms detected. It was observed that the group containing sAD samples and samples from familial AD cases with Presenilin mutations aggregated faster than the group containing sAD cases and familial AD cases with APP mutations respectively [34]. Their results, in combination with ours, show that the changes in aggregation kinetics reflect directly on the seeding capabilities of Aβ extracted from distinct clinical variants. In the case of rpAD, it is probable that a longer lag phase reflects the presence of oligomeric species for a longer duration in the brain. This delay in the generation of mature fibrils and plaques, which is a protective physiological measure to prevent Aβ toxicity, raises red flags (Fig. 5 e and f). Collectively, these changes may lead to more neurodegeneration in rpAD brains in comparison to sAD brains.

Although the aggregation of rpAD fibrils appeared to be slower than sAD fibrils, they featured the presence of larger and more polymorphous aggregates in comparison to sAD cases at the end of the reaction. sAD-derived reactions, on the other hand, had regular well-defined fibrils. Previous studies have also attributed these differences to alterations in inherent charges of the substrate, hydrophobicity, and the capability of proteins to generate secondary structures required for nucleation [35]. As the reactions were conducted using the same substrate, and our preliminary infrared spectroscopy experiments detected no variations within the secondary structures (data not included), the structures observed for rpAD may be products of highly hydrophobic fibrils that have a greater propensity to bind with each other. Moreover, as secondary nucleation is dependent on the availability of fibril surface, and rpAD-derived fibrils are buried within larger structures, these results also explain why rpAD cases reached the stationary phase at a lower absorbance in comparison to sAD cases.

We also recognized cofactors, specifically those proteinaceous in nature, to further elucidate the reasons underlying the differences in RT-QuIC profiles of sAD and rpAD brains. A majority of proteins identified in this dataset were cytoplasmic proteins that have been previously reported to undergo changes in solubility in response to Aβ pathology, leading to their isolation from the insoluble fraction, rather than the soluble fraction, of the brain proteome [36]. The analysis was therefore focused on the targets that have previously been associated with assisting or inhibiting the fibril formation of prions or prion-like proteins, or that have the capability to cross seed Aβ and implicate amyloid formation [37–40]. The heterogeneity among the clusters of proteins that impact amyloidogenesis directly provides an insight into the environment of fibrils in the brain. The proteins involved in promoting fibril formation were highly enriched in rpAD cases whereas sAD cases presented a higher number of proteins that can cross seed Aβ. Collectively, these changes may affect the amyloid formation and contribute towards the discrepancies observed in kinetic curves. This list of putative accessory proteins is being validated by seeding all common and sub-type specific Aβ under different concentrations of the accessory proteins.

The structure of aggregates is closely associated with their toxicity mechanisms [41]. However, in their final conformation, Aβ fibrils from sAD and rpAD presented no significant differences in toxicity in vitro. We then analyzed Aβ interactions in the brain to establish if there are differences in the way they impart toxicity in sAD and rpAD. The study of human samples conducted using co-IP identified putative interactors and provided an insight into different functional pathways modulated by tris-soluble pools of Aβ. Several known regulators of signaling pathways, including serine/threonine-protein phosphatase PGAM5, GTPase KRas, and peroxiredoxin, were seen to selectively interact with Aβ in rpAD, but not sAD, brains [42, 43]. Aβ was also observed to interact with voltage-gated potassium channels in all experimental groups. However, its higher expression in sAD and rpAD may result in greater dose-dependent impairment of potassium channels and may also trigger a greater disturbance in neurotransmission [44, 45]. Additionally, synaptotagmin-1 was detected in rpAD cases but not in sAD cases. Synaptotagmin-1 is keenly involved in the release of neurotransmitters through its interactions with the SNARE complex and phospholipid membranes, and it has previously been reported to be increased in Alzheimer’s disease–associated pathologies [46, 47].

Our study covered several stages of Aβ pathology from synthesis to fibrillization and interaction. Although it has pointed out several similarities and differences among the targeted clinical variants of AD, it is important to acknowledge certain limitations. Firstly, the amount of brain samples required from the mentioned patients to further our hydrophobicity and toxicity analysis hindered us. We were unable to include data from hydrophobicity interaction chromatography and toxicity assays with more potent, and purified, monomeric, and oligomeric species of Aβ. We are now in the process of acquiring more material to further our findings and establish Aβ RT-QuIC as the important tool to define clinical subtypes of AD. Secondly, Aβ profiles are sensitive to brain regions, extraction methods, purification techniques, and mass spectrometric technology employed. We chose the methodology that has worked best for those in the field before and provided analyzable results for us. However, doing an IP with other antibodies or using a different mass spectrometric approach may bring forward proteoforms that have been missed in this study. We are already moving forward in this regard and attempting to optimize in situ identification that will detect all the proteoforms lost during extraction and purification. Furthermore, the current experiments have been optimized with Aβ_40_ and Aβ_42_ but it is equally important to establish RT-QuIC profiles for the other proteoforms detected in the MALDI-MS dataset. These results will establish the basis for testing other proteoforms in different ratios to test their wider impact on the prognosis of AD.

## Conclusion

We investigated multiple aspects of Aβ biology to characterize slow and rapid variants of Alzheimer’s disease. Our results present a signature of 33 distinct C and N-terminally truncated pathophysiological proteoforms, including the commonly targeted Aβ_40_, Aβ_42_, Aβ_4-42_, Aβ_11-42_ that are at play in sAD and rpAD brains. Although their signature does not appear to define the prognosis of AD, their increased synthesis in rpAD brains (as indicated by significantly higher β-secretase to α-secretase ratio and slightly elevated AβTotal) followed by slower, yet more potent (in terms of the size of products generated) aggregation, and differential interactions present some promising targets for further analysis. In the light of these preliminary findings, it can be postulated that although the fibrils generated by rpAD brains appear to be capable of generating larger amorphous aggregates, their conversion from seeds to fibrils is slower. During this process, Aβ may exist as more toxic oligomeric species for a longer duration and impart greater toxicity on surrounding neurons. The clinical phenotype resulting from these changes may, therefore, present a faster rate of progression even though the overall profiles of total Aβ in CSF and brain appear highly similar. Collectively, this evidence supports the differences in aggregation propensities may underlie the atypical progression of AD.

## Supplementary Information

Below is the link to the electronic supplementary material.

Supplementary file1 (DOCX 758 KB)

## Data Availability

The datasets supporting the conclusions of this article are included within the article and as its additional files. All raw data is available from the corresponding author upon request.
